# Targeted Deletion of *Los1* Homologue Affects the Production of a Recombinant Model Protein in *Pichia pastoris*

**DOI:** 10.52547/ibj.25.4.255

**Published:** 2021-05-15

**Authors:** Najmeh Zarei, Hosnieh Ghasemi, Mahsa Nayebhashemi, Mozhgan Zahmatkesh, Monire Jamalkhah, Nafiseh Moeinian, Zahra Mohammadi, Somayeh Enayati, Vahid Khalaj

**Affiliations:** 1 Medical Biotechnology Department, Biotechnology Research Center, Pasteur Institute of Iran, Tehran, Iran;; 2 Department of Biotechnology, College of Science, University of Tehran, Iran

**Keywords:** Aging, Longevity, * Pichia pastoris*, Recombinant proteins

## Abstract

**Background:**

The methylotrophic yeast *Pichia pastoris* is an appealing production host for a variety of recombinant proteins, including biologics. In this sense, various genetic- and non-genetic-based techniques have been implemented to improve the production efficiency of this expression platform*. Los1 *(loss of supression) encodes a non-essential nuclear tRNA exporter in *Saccharomyces cerevisiae*, which its deletion extends RLS. Herein, a *los1*-deficient strain of *P. pastoris* was generated and characterized.

**Methods:**

A gene disruption cassette was prepared and transformed into an anti-CD22-expressing strain of *P. pastoris*. A *δ los1 *mutant was isolated and confirmed. The drug sensitivity of the mutant was also assessed. The growth pattern and the level of anti-CD22 ScFv expression were compared between the parent and mutant strains.

**Results:**

The *los1* homologue was found to be a non-essential gene in *P. pastoris*. Furthermore, the susceptibility of *los1* deletion strain to protein synthesis inhibitors was altered. This strain showed an approximately 1.85-fold increase in the extracellular level of anti-CD22 scFv (*p* < 0.05). The maximum concentrations of total proteins secreted by *δ los1* and parent strains were 125 mg/L and 68 mg/L, respectively.

**Conclusion:**

The presented data suggest that the targeted disruption of *los1* homologue *in P. pastoris* can result in a higher expression level of our target protein. Findings of this study may improve the current strategies used in optimizing the productivity of recombinant *P. pastoris* strains.

## INTRODUCTION

The methylotrophic yeast *Pichia pastoris* is frequently used for the high level production of recombinant proteins, especially where the *Escherichia coli *system fails to deliver correctly folded functional proteins, or the *Saccharomyces cerevisiae* system produces hyper-mannosylated inactive proteins^[^^1^^]^. The recent development of glycoengineered *Pichia* strains generating human-like glycan pattern, along with the adaptation of new genome editing tools, such as CRISPR system, has offered further advantages for the industrial application of *Pichia* platform^[^^2^^,^^3^^]^.

Available reports indicate the highest amount of intracellular (22 g/L) and extracellular (18 g/L) protein expression levels in *P. pastoris*^[^^4^^]^. As a matter of fact, not all proteins are produced at such a high level, and the amount of protein expression depends largely on the intrinsic properties of the target protein. Fortunately, in many cases, the initial expression levels can greatly be improved by the manipulation of host genome or culture conditions. The genetic manipulation of host cells using targeted gene deletion or overexpression has been one of the most important optimization approaches that can affect the quantity and quality of a desired protein product^[^^5^^]^. 

The production of recombinant proteins in *P. pastoris *is a growth-dependent process^[^^6^^]^ such that host cell engineering for cell lifespan extension is considered as a promising strategy in increasing the yield of recombinant protein production. Cell aging can affect both protein synthesis and degradation, by altering the components of the translation machinery, as well as the intracellular distribution of newly synthesized proteins^[^^7^^,^^8^^]^. In this sense, the manipulation of aging process may alter the final yield of recombinant products during the mass production procedures.

The budding yeast, *S. cerevisiae*, has been an excellent model for aging and lifespan-related studies. Both RLS (the number of daughter cells generated by a mother cell before replicative senescence) and CLS (the survival time of a yeast cell in non-dividing condition) have already been defined, and molecular mechanisms involved in the yeast lifespan have been investigated in details^[^^9^^,^^10^^]^. So far, several genes identified in yeast have been shown to be able to modulate the aging process through different mechanisms^[^^11^^,^^12^^]^. *Los1* (loss of supression), as a non-essential gene in *S. cerevisiae*, encodes a nuclear tRNA exporter, and its deletion robustly increases lifespan in this organism^[^^12^^]^. As a member of the conserved β-importin family, *Los1* dictates the direction of tRNA transport across nuclear pores using the nuclear-cytoplasmic gradient of RanGTP^[^^13^^]^. Considering the conserved mechanisms influencing the lifespan and aging among eukaryotes^[^^12^^,^^14^^,^^15^^]^, herein, we report the inactivation of *Los1* gene homologue, in *P. pastoris* using a site-directed disruption construct. The impact of this deletion on the viability and growth of the deletant was monitored, and the production yield of a model recombinant protein, anti-CD22 scFv, was investigated.

## MATERIALS AND METHODS

**Strains, plasmids, and culture conditions**

**Fig. 1:**
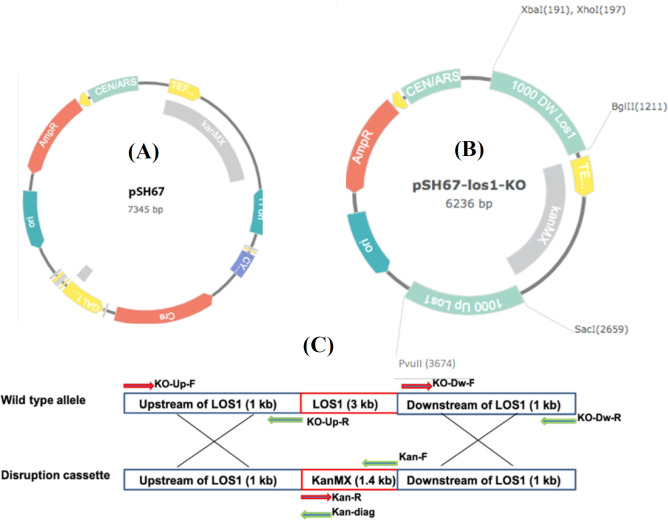
Schematic diagram of the various constructs used in the study. (A) pSH67, (B) pSH67-Los1-KO, and (C) replacement of *Los1* gene by KanMX through the homologous recombination. The positions of primers used in the preparation of the disruption cassette or confirmatory PCRs are shown by arrows

*E. coli* Top 10F′ was employed as a host for DNA manipulations, and *P. pastoris* strain GS115 (his4) with mut^+^ phenotype (Invitrogen, San Diego, CA, USA) was used for protein expression studies. pGEM-T Easy Vector (Promega, Madison, USA) was applied for cloning of PCR products, and the pSH67 plasmid, carrying G418 resistance gene (KanMX), as a yeast selection marker, was utilized as an intermediate vector for assembling the *los1 *disruption cassette (Fig. 1). *E. coli* Top 10F′ was cultured in Luria-Bertani medium (in w/v; 1% tryptone, 0.5% yeast extract, and 1% NaCl, pH 7.0) at 37 °C. Ampicillin was added to the LB medium for plasmid selection at the final concentration of 100 μg/ml. The yeast *P. pastoris* strain was grown in YPD medium (in w/v; 1% yeast extract, 2% peptone, and 2% dextrose), whereas YPD plates containing G418 (300 μg/mL or as indicated) were used for the selection of G418-positive *P. pastoris* transformants. The BMGY was prepared with 2% peptone, 1% yeast extract, 1% glycerol, 1.34% yeast nitrogen base, with ammonium sulfate but without amino acids, and 4 × 10^-5^% biotin in 100 mM potassium phosphate buffer. The preparation of BMMY was carried out the same as BMGY, except for the replacement of glycerol with 0.5 % methanol. 

**Identification of** ***Los1*** **homologue in** ***P. pastoris***

For the identification of the *Los1* homologue in *P. pastoris*, the amino acid sequence of *S. cerevisiae* *Los1 *was retrieved from the UniProt database, and a protein blast against fungal database was performed (https://www.uniprot.org/). Similar searches were also carried out at http://blast.ncbi.nlm.nih.gov and http://pichiagenome-ext.boku.ac.at/. Phylogenomic databases such as eggNOG (http://eggnogdb.embl.de), HOGENOM (http://hogenom.univ-lyon1.fr/), OrthoDB (https://www.orthodb.org/) and Inparanoid (http:// inparanoid.sbc.su.se/cgi-bin/index.cgi) were also used to investigate the presence of the *Los1* homologue in *P. pastoris*. Nuclear localization signal sequence was predicted using an online software (http://mleg.cse.sc. edu/seqNLS/).

**Preparation of** ***Los1*** **gene disruption cassette**

The target gene disruption construct was prepared using standard PCR and cloning protocols, and the final cassette was validated by restriction mapping and DNA sequencing^[^^16^^]^. Briefly, to facilitate homologous recombination, a 1000-bp upstream and a 1000-bp downstream flanking regions of *Los1* homologue in *P. pastoris *were amplified using specific primer sets KO-UP_F/R and KO_DW_F/R, harboring *Sac*I/*Pvu*II and *Xba*I/*Bgl*II restriction sites, respectively (Table 1). The amplified fragments were sequentially subcloned into pSH67, the upstream and downstream of KanMX selection marker, to create the pSH67-*los1*-KO construct (Fig. 1A and 1B).

**Transformation of** ***P. pastoris*** **and selection of disruptant clones**

The pSH67-*Los1*-KO construct was double-digested with *Xho*I*/Pvu*II enzymes, and the linearized *Los1*-KO fragment, containing KanMX and flanking *los1* up and down sequences, was purified from agarose gel and used to transform the competent *P. pastoris GS115* strain, expressing scFv, according to the Invitrogen protocol. Briefly, the competent *P. pastoris GS115 * cells were prepared by successive cultivation in YPD broth medium, followed by sequential washing steps using ice-cold water and sorbitol. The *los1*-KO fragment (5 µg in 10 μL of dH_2_O) was mixed with 80 μL of competent cells and subjected to electroporation, using a Gene Pulser Xcell™ device (Biorad, USA; settings: 1,500 charging voltage, 25-μF capacitance, and 200-Ω resistance). G418-resistant colonies were selected on YPD agar medium containing 300 μg/mL of G418 at 30 °C. Next, the large transformant colonies were purified by re-streaking on YPD plates, containing 300 μg/mL of G418. The selected transformants were analyzed for the occurrence of homologous recombination through diagnostic PCRs using different specific primer sets. To design these primers, we selected a sequence from the *Pichia *genome, suited at the 30-bp upstream of the UP fragment, used in the KO construct (1030_UP_Los, Table 1). This sequence was absent from the *los1*-KO construct and was thus used as the forward primer. A primer targeting the 5’ end of the KanMX gene (Kan_F, Table 1) was used as the reverse primer, for the first round of screening. For further confirmation, the second diagnostic PCR was performed using a primer designed for the 100-bp region, at the 3’ end of the KanMX gene (Kanـdiag, Table 1) and primer 1030_UP_Los. The correct deletion transformants were designated as *δ los1*GS115/scFv.

**Table 1:** Primers used in this study

**Primer**	**Sequence**
KO_UP_F	CAGCTG_ATAGCACCTTCCTCGTTC
KO_UP_R	GAGCTC_TAGCAGTGGGTGGTGAACT
	
KO_DW_F	AGATCT_GTGACTTATGAGAATAAGT
KO_DW_R	TCTAGA_AGATGCGCTTTCAAGCAA
	
Kan_F	GACATGGAGGCCCAGAATAC
Kan_R	CAGTATAGCGACCAGCA
	
1030_UP_Los	CTTCCAGCCTTAACCTTGC
Kan-diag	TCGATTCGATACTAACGCC

Restriction site sequences are underlined.

**Preparation of growth curves**

For investigating the effect of *los1* inactivation on the growth of different strains used in this study, a single colony of GS115, GS115/scFv, and *δ los1*GS115/scFv was picked and grown in 5 ml of YPD. The cultivation was started with an OD_600_ of 1.0, and sampling and OD determination were performed at the following time: 12, 24, 48, 72, 96, 120, 144, and 168 h. The same protocol was carried out in BMMY expression culture medium. 

**Evaluation of cell viability using trypan blue test**

For more accurate determination of growth patterns, in addition to OD measurements, the number of live cells was counted using trypan blue staining method, followed by light microscopy observation at specific time points of 12, 24, 48, 72, 96, 120, 144, and 168 hours.

**Determination of** **chemical sensitivity**

The *in vitro* susceptibility of *δ los1*GS115/scFv to some antifungal agents (hygromycin, itraconazole, nystatin), as well as protein synthesis affecting compounds, rapamycin and cycloheximide, was assessed by measuring MICs based on the Clinical and Laboratory Standards Institute Guidelines, with some modifications^[^^17^^]^. Briefly, a total of 10^3^ yeast cells were inoculated onto each well of a 96-well microtiter plate containing RPMI-1640 medium, enriched with 2% glucose and buffered to pH 7.0 with 3-(N-morpholino)propanesulfonic acid. The plates were incubated at 30 ^o^C, and MICs were assessed at a final compound concentration range of 0-200 µg/mL after 24 and 48 hours.

**Expression of anti-CD22 scFv in** ***δ los1*** **GS115/scFv strain**

Single colonies of *los1* deletion strain were grown individually in 25 mL of BMGY medium at 30 °C, 250 rpm for 16–18 h to reach an OD_600_ of 2 to 6 (log phase growth). Thereafter, the cells were harvested by centrifugation at 3,000 ×g at room temperature for 5 min. To induce protein expression, the pellets were resuspended in 100–200 mL of BMMY medium to an OD_600_ of 1.0 and grown at 30 °C, while shaking at 250 rpm. Fresh methanol (100%) was added, every 24 h, to a concentration of 0.5% to compensate for losses from evaporation and metabolism. The medium (25 mL) from each culture was harvested every 24 h and centrifuged 5800 ×g at 4 °C for 10 min. To detect the recombinant protein, the culture supernatants were concentrated using an Amicon® Stirred Cell (Merck, USA) and then analyzed by SDS-PAGE and Western blotting. 

**SDS-PAGE and Western blot analysis**

Culture supernatant samples were analyzed by electrophoresis on a 12% polyacrylamide gel according to the current protocols. The gels were stained with Coomassie Blue R-250, and the protein lanes were scanned, and the expression levels of the target scFv were evaluated through the intensity measurements of protein bands using a gel image analysis software (Quantity One^TM^, ver. 4.6.3; Bio-Rad Laboratories, Hercules, CA, USA). Western blot analysis was performed in accordance with standard protocols^[^^16^^] ^using a mouse horse-radish peroxidase-labeled monoclonal antibody against the 6× His-tag (Invitrogen). Finally, positive bands were visualized using an enhanced chemiluminescence detection system (Amersham Life Sciences, Buckinghamshire, UK). In each experiment, the total amount of extracellular proteins was assessed by standard Bradford method. 

**Comparison of anti-CD22 scFv expression at different times in GS115/scFv and** ***δ los1*** **GS115/scFv strains**

 In order to compare the expression levels of anti-CD22 scFv in mutant and parental strains, we induced expression in GS115/scFv and *δ los1*GS115/scFv single clones, as per the Invitrogen protocol under controlled conditions. The cells were grown in BMGY medium overnight and then transferred to BMMY medium (150 ml) with the same OD = 1.0 and incubated under exactly the same conditions. At specific time points (12, 24, 48, 72, 96, 120, 144, and 168 h) after incubation, culture sampling was performed. The supernatant was separated by centrifugation at 5800 ×g at 4 °C for 10 min. Equal volumes of supernatants from both clones were analyzed by SDS-PAGE, and the resolved proteins were visualized by Coomassie Blue Staining. The relative amounts of the target recombinant scFv were determined through the densitometry analysis of SDS-PAGE gels.

**Statistical analysis**

The results were expressed as mean ± SD (n = 2). Data were analyzed by double-tail Student’s t-test. The differences were considered statistically significant at *p* < 0.05.

## RESULTS

**Sequence analysis of** ***P. pastoris los1*** **homologue**

*Los1* sequence from *S. cerevisiae* (Uniprot: P33418) was compared with the *P. pastoris* genome using BLASTp (https://blast.ncbi.nlm.nih.gov/Blast.cgi), and the top scoring match (Protein ID:XP_002491537.1) was selected for further analysis. This open reading frame, annotated as Exportin-T in UniProt (C4R183_KOMPG) and KEGG (K14288) databases, contains 1071 amino acids, with a theoretical pI of 5.5 and a molecular weight of ~123 kDa. Nuclear localization signal prediction identified the residues 21-64 as the potential nuclear targeting signal. The *Los1*-encoding sequence in *Pichia* genome is located on chromosome 2 (NC_012964.1 nucleotides 1,157,057..1,160,334) and contains 3216 nucleotides.

**Fig. 2:**
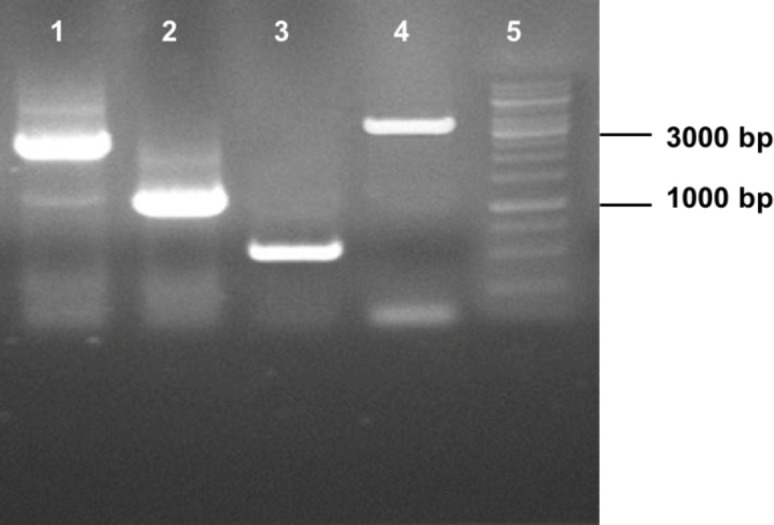
Confirmatory PCRs on the genomic DNA of *los1* disruption mutant of *P. pastoris*. Lane 1, PCR product amplified by 1030_UP/ Kan_F primer set (2500 bp); lane 2, PCR product with 1030_UP/Kan-diag primers (1100 bp); lane 3, PCR product with actin primers (as a control; 500 bp); lane 4, PCR with 1030_UP/KO_DW_R primers amplified the disruption construct as a 3.6-kb fragment due to the insertion of KanMX; lane 5, DNA marker

***Los1*** **homologue as a non-essential gene in** ***P. pastoris***

The *los1*-KO fragment was successfully assembled in pSH67 and confirmed by restriction mapping and sequencing (data not shown). Transformation of *P. pastoris* strain by *los1*-KO construct resulted in several G418-resistant colonies. Colony PCR screening was carried out on 26 transformants using designed specific primers (Table 1). A successful gene disruption event was verified by PCR amplification of a 2500-bp fragment, with 1030_UP_Los/Kan_F primers (Fig. 2, lane 1). This event was further confirmed by the second PCR using Kan-diag/1030_UP_Los primers, which resulted in a 1100-bp PCR product (Fig. 2, lane 2) Also, in the case of correct replacement (gene disruption**, **Fig. 1C), a 3600-bp fragment was expected to be amplified by the 1030_UP_Los/KO_DW_R primers in the disrupted genome, as opposed to a 4600-bp fragment, in the parent strain (Fig. 2, lane 4). Five *los1* gen-disrupted strains were isolated via the above screening steps, and all were able to grow normally on YPD agar, indicating that the *los1* gene is non-essential for the viability of *P. pastoris*.

**Effect of** ***los1*** **inactivation on the growth curve of** **GS115/scFv**

**Fig. 3:**
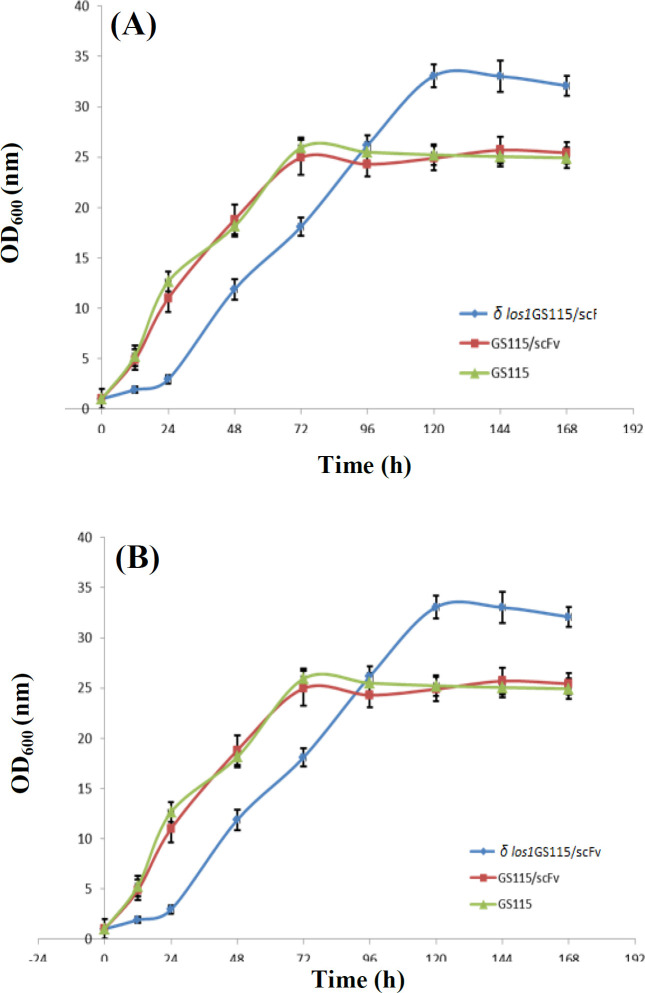
Growth curve of *δ* *los1*GS115/scFv compared to GS115/scFv and GS115 in (A) YPD and (B) BMMY media. X-axis represents sampling intervals of 24 hours

The growth pattern of GS115/scFv and *δ* *los1*GS115/scFv strains were examined and compared to the wild type GS115, in both YPD and BMMY media. The results demonstrated that both examined strains followed a similar sigmoidal pattern of growth in the two media. However, as shown in Figures 3, the initial growth rate of the *δ* *los1*GS115/scFv was slower than that of the parental GS115/scFv and wild type GS115 strains. In this regard, the final OD achieved by the *δ* *los1*GS115/scFv strain was higher than the other two strains. In YPD medium, maximum OD_600_ of about 25 was reached at 72 h, while the maximum OD_600_ for *δ los1*GS115/scFv was about 33 at 120 h. The same trend was observed during the growth in BMMY medium. The maximum ODs for GS115/scFv, GS115, and *δ* *los1*GS115/scFv clones in BMMY were 22, 23, and 29, respectively.

**Effect of** ***Los1*** **disruption on the viability of GS115**/**scFv strain**

**Fig. 4:**
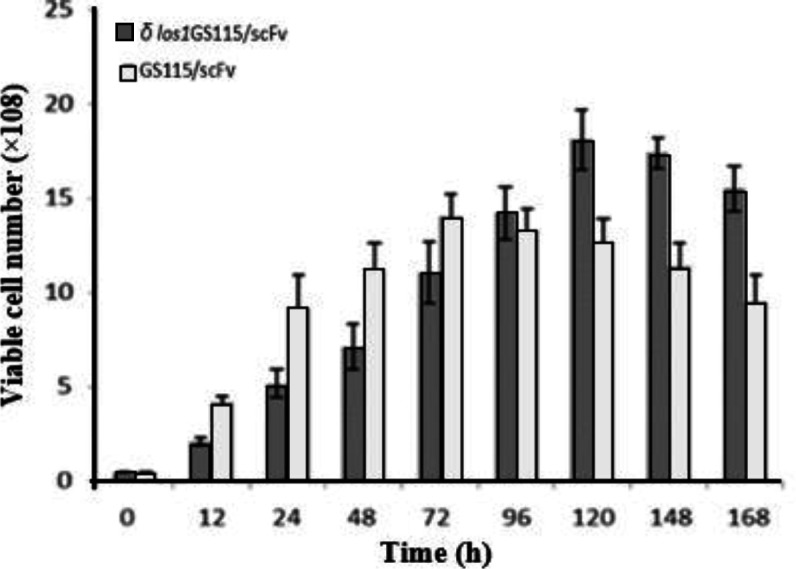
Evaluation of viability of *δ* *los1*GS115/scFv in comparison to GS115/scFv cells

The bar chart in Figure 4 shows the effect of *los1* disruption on the viability of the cells. These results indicate an increase in the number of viable *δ los1*GS115/scFv up to 120 hours and GS115/scFv up to 72 hours of growth, followed by an approximately 15% decrease per every 24 h. The number of live *δ los1*GS115/scFv cells was less than that of GS115/scFv in the first 72 hours. Subsequently, at 96 h, a sharp rise was observed in the number of viable *δ los1*GS115/scFv cells compared to the parental GS115/scFv, which was followed by a general increase in the number of live* δ* *los1*GS115/scFv cells, thereafter.

**Altered chemical susceptibility of δ** ***los1*GS115/scFv**

Although we did not observe any significant differences in MICs measured for several antifungal agents, including hygromycin, itraconazole, and nystatin, the susceptibility to rapamycin and cycloheximide was markedly different between *δ los1*GS115/scFv and the parent strain such that the *los1 *mutant strain was more sensitive to rapamycin (MIC = 0.62 µg/ml) as compared to the parent strain (MIC = 2.5 µg/ml) and more resistant to cycloheximide (MIC of 50 µg/ml *vs. *25 µg/ml). 

**Expression of anti-CD22 by** ***δ*** ***los1*** **GS115/scFv**

**Fig. 5:**
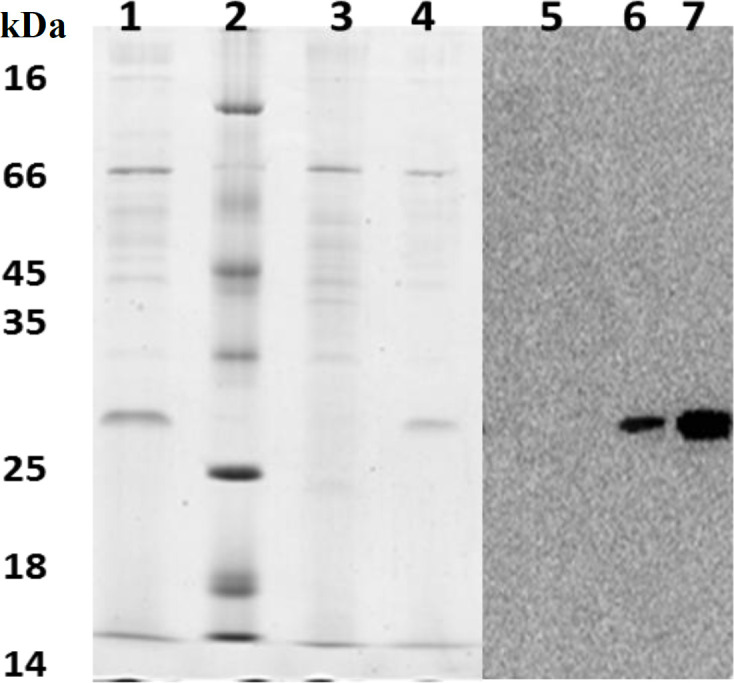
Analysis of proteins presented in the culture supernatants. Lanes 1-4, SDS-PAGE analysis; lane 1, protein bands of *δ* *los1*GS115/scFv culture supernatant; lane 2, protein marker; lane 3, supernatant of GS115 culture; lane 4, supernatant of GS115/scFv; lane 5, 6, and 7 indicate Western blot analysis on the supernatant of GS115, GS115/scFv, and *δ* *los1*GS115/scFv, respectively. Lanes 6 and 7 show positive signals with the expected size of ~30 kDa

A single colony of *δ* *los1 P. pastoris* was selected, and the expression of the anti-CD22 scFv was induced by methanol as described above. The 72-h culture supernatant was collected, concentrated and analyzed by SDS-PAGE. A single protein band around 30 kDa was clearly observed in the culture medium of GS115/scFv and δ *los1*GS115/scFv, while the same band was absent from the culture supernatant of GS115 cells, transfected with the control plasmid (pPICZαA; Fig. 5). To confirm the expression of scFv, cell-free supernatants were further analyzed by Western blot analysis using mouse anti-6× His-tag antibody. The results demonstrated that anti-6×-His antibody positively reacted with a protein band of similar molecular weight observed in SDS-PAGE. Again, no similar bands were detected in the negative control (Fig. 5). These findings showed that the anti-CD22 scFv was successfully expressed in the recombinant *δ* *los1*GS115/scFv strain, and *los1* inactivation did not interfere with anti-CD22 scFv production in *P. pastoris*.

***Effect of Los1*** **gene disruption on anti-CD22 scFv expression levels**

**Fig. 6:**
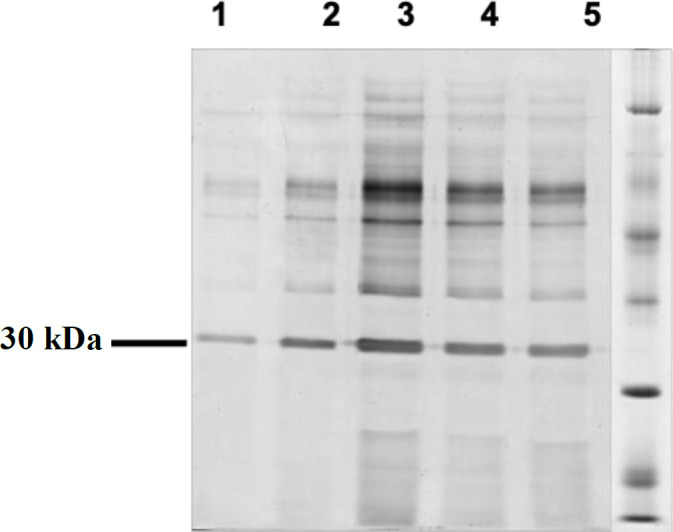
SDS-PAGE analysis of culture supernatant of *δ* *los1*GS115/scFv after 72 h (1), 96 h (2), 120 h (3), 144 h (4), and 168 h, and (5) protein bands of ~30 kDa indicating the expression of recombinant anti-CD22 scFv

**Fig. 7:**
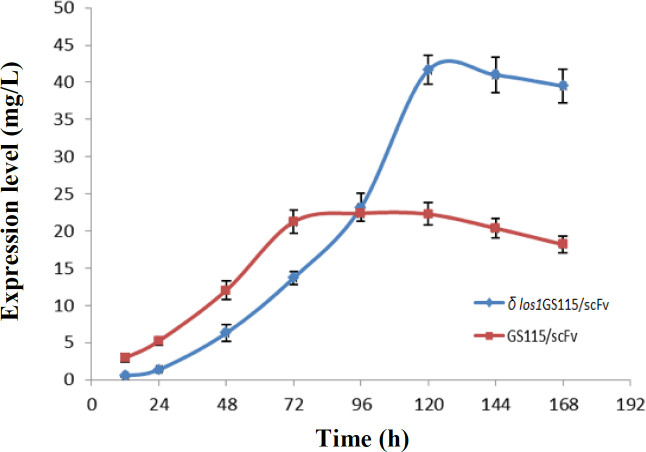
Evaluation of anti-CD22 scFv expression levels at different times (every 24 h) in *δ* *los1*GS115/scFv and GS115/scFv

**Fig. 8:**
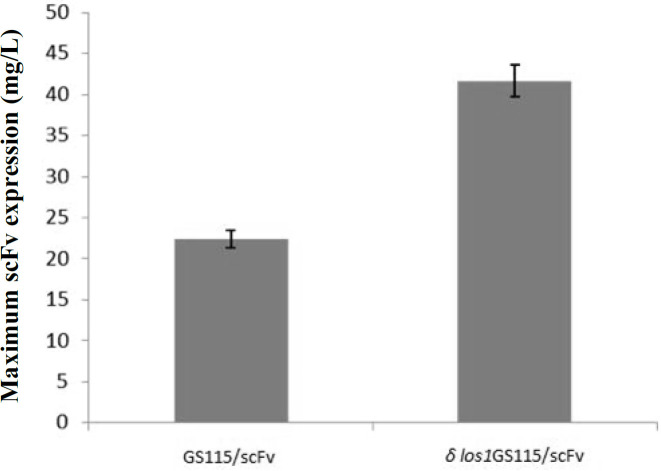
The maximum concentration of anti-CD22 scFv expressed by *δ* *los1*GS115/scFv compared to GS115/scFv

**Fig. 9:**
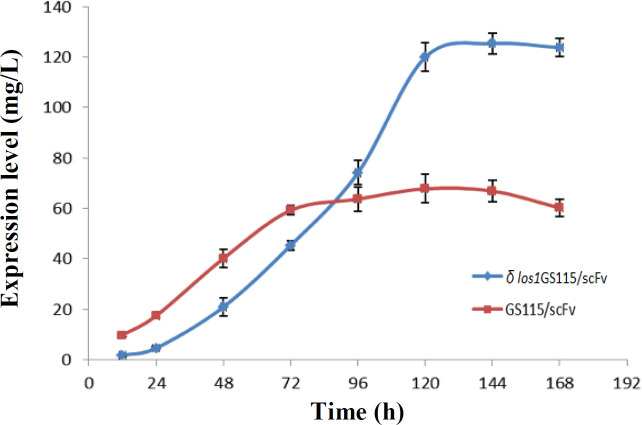
Evaluation of total protein concentration expression at different times (every 24 h) in *δ* *los1*GS115/scFv and GS115/scFv

**Fig. 10:**
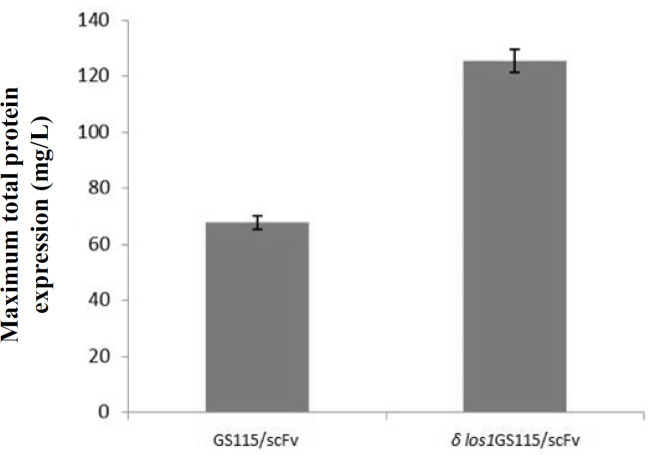
The maximum total extracellular protein concentration produced by *δ* *los1*GS115/scFv compared to GS115/scFv

The increase in the extracellular production of anti-CD22 scFv over time was clearly found in both *δ* *los1 *and parent GS115/scFv strains, except for *δ los1*GS115/scFv clone, where the expression levels started to increase slowly during the first 72 hours and then continued with a steeper slope (Figs. 6 and 7). In the GS115/scFv clone, the highest level of extracellular anti-CD22 scFv appeared at 72 hours and then gradually declined. However, in *δ* *los1*GS115/scFv clone, the peak expression of the secreted scFv was achieved at 120 hours, which declined thereafter. As depicted in Figure 8, the *δ* *los1*GS115/scFv strain showed about 1.85-fold increase in the extracellular level of anti-CD22 scFv, compared to the parent GS115/scFv strain (*p* < 0.05). A similar pattern was also observed for total secreted proteins (Fig. 9). The maximum concentrations of total proteins secreted by *δ* *los1*GS115/scFv and GS115/scFv strain were 125 mg/L and 68 mg/L, respectively (Fig. 10). 

## DISCUSSION

Over the last decades, *P. pastoris* has emerged as a highly popular expression system for the recombinant production of various heterologous proteins^[^^18^^]^. More than 700 recombinant proteins have so far been expressed in *P. pastoris*, and more than 70 protein products are in the final stages of research before entering the market^[^^19^^]^. Available reports indicate that* P. pastoris* is able to produce up to 20-30 g/L of recombinant proteins^[^^4^^]^. The ability of this organism to produce high amounts of protein, as w ell as the FDA approval for certain biopharmaceutical products derived from this yeast system, has made *P. pastoris* a valuable host for industrial-scale heterologous protein production^[^^2^^]^. 

Despite the impressive advantages, some degrees of process optimization are required to achieve the maximum yield of the target proteins, using the Pichia platform. The recombinant protein production in *P. pastoris* is a growth-dependent process^[^^6^^]^. In this regard, running the production process under the high cell density condition can result in increased volumetric productivity^[^^20^^]^. Meanwhile, viable cell volume, as a fundamental bioprocess parameter, has been the subject of different studies, as any alteration in this variable can affect the quality and quantity of the final product^[^^21^^-^^23^^]^. To this end, strategies leading to the extension of the lifespan or delay in the aging process may effectively increase the viable cell densities. Up to now, many environmental and genetic factors are known that affect the RLS in yeast. Some well-studied interventions, like dietary restrictions, as well as mutations mimicking low nutrient availability^[^^8^^,^^24^^]^, deletion of some UPR target genes^[^^8^^]^, and reduced target of rapamycin (TOR) and protein kinase A signaling^[^^25^^]^, have been effective in lifespan modulations. Further to the above points, many studies have demonstrated the relationship between lifespan (particularly RLS), aging, and protein production^[^^8^^,^^26^^,^^27^^]^. The effectiveness of proteostasis control systems, which regulate the production and quality of the proteins in the cell, is diminished with age, giving rise to the accumulation of damaged proteins, which in turn inhibit cell functionality and efficient protein production^[^^28^^]^. Anisimova *et al.*^[^^8^^]^ have clearly discussed that every step in the protein lifecycle, most notably protein synthesis and degradation, is relevant to the aging process. Several studies have demonstrated that the overall level of protein production is reduced with age^[^^8^^,^^28^^,^^29^^]^. Other investigations highlighted the reduced ribosome abundance, as well as attenuated activity and levels of major initiation and elongation factors^[^^30^^,^^31^^]^. Aging can also affect the ability of cells in controlling the intracellular distribution of newly synthesized proteins^[^^8^^]^. Considering the negative effects of aging on the yield and quality of protein products in the cell, in the present study, we investigated the effect of *los1* gene inactivation on the anti-CD22 scFv production level, in *P. pastoris*.

*Los1* is supposed to be the main exportin for pre-tRNA-containing intron in yeast. Although the efficient transfer of tRNAs from the nucleus to the cytoplasm is vital for protein synthesis, *los1* is not essential in yeast *S. cerevisiae*, and its deletion increases the yeast life span by 60%^[^^12^^]^. Consistent with data available for *S. cerevisiae* and some other fungal species^[^^32^^,^^33^^]^, the Pichia *los1* homologue was found to be a non-essential gene, indicating the existence of a functional redundancy in tRNA export in *P. pastoris*. It has been well documented that following the inactivation of *los1*, the nuclear retention of tRNA autonomously signals the activation of several pathways, leading to the extension of lifespan^[^^12^^]^.

In the present study, we observed a slower growth rate for *δ* *los1*GS115/scFv in comparison to the GS115/scFv, which in turn resulted in decreased levels of protein expression. This slow behavior may be due to the tRNAs limitations, which can reduce protein translation and growth rates in the early stages of the production process^[^^34^^]^. However, after 72 h, we observed a sudden increase in the growth rate, deduced from the OD values of culture media. The anti-CD22 scFv protein expression levels, produced by the *δ los1*GS115/scFv strain at different times, also followed a similar pattern to the growth curve with a final yield of ~1.85-fold higher than that of the parent strain. Since the amount of productivity in yeast cells is a function of cell density and specific production rate, the absence of *los1* activity can enhance the final production yield via increasing the viability and subsequently, cell density.

Buchetics *et al*.^[^^35^^]^ have reported that the over-expression of the B-type cyclin CLB2 in *P. pastoris* shifted the cell population to the G2/M phase, in which a higher proportion of cells are in the budding state. This manipulation led to higher secretion efficiencies of different recombinant proteins, even at lower growth rates, where the wild-type cells usually show low secretion rates. As the *los1* deletion in yeast results in increasing RLS (the number of buds a mother can produce before replication ceases), it can be speculated that the absence of *los1* activity may increase the budding state of *δlos1*GS115/scFv cells, which in turn causes the higher extracellular production of the target protein over time. Moreover, Puxbaum and colleagues^[^^36^^]^ have recently shown that the bud is a specific site for the protein secretion, and those manipulations, which keep more cells in the bud state, promotes a higher secretion efficiency.

To test whether the deletion of *los1 *has any effect on the chemical susceptibility of the yeast, MIC levels were determined for different compounds. Among them, the *los1* disruption strain was more sensitive to rapamycin, in comparison to the parental strain. Rapamycin has now emerged as an attractive therapeutic strategy with immunosuppressant properties. In addition, rapamycin has been proposed as a calorie restriction mimetic to extend the lifespan of various organisms. These rapamycin actions are mediated through the inhibition of the mechanistic target of rapamycin kinase and its downstream signaling pathways^[^^37^^]^. Doi et al.^[^^38^^]^ carried out a chemical genomics screen for rapamycin-sensitive mutants using the genome deletion library, which covers 95.3% of all non-essential fission yeast genes and confirmed 59 mutants to be rapamycin sensitive. 

Among these mutants, *los1*-mutants scored as moderately sensitive to rapamycin, an observation that is consistent with our results. Cycloheximide is a potent inhibitor of protein synthesis, which acts through binding the 60S ribosomal subunit and arresting protein translation^[^^39^^,^^40^^]^. In this study, we found that *δ* *los1*GS115/scFv was more resistant to cycloheximide, as compared with the wild type strain. This resistance can be due to the reduced activity of protein translation machinery in *los1* disruptant, making it less sensitive to the inhibitory effects of cycloheximide.

In conclusion, we demonstrated that *los1* homologue is not essential for the viability of the yeast *P. pastoris*. However, the targeted disruption of this gene caused a higher expression level of our protein model. Our findings may serve the recombinant protein industry, where *P. pastoris* is used as a production host. 

## References

[1] Ma Y, Lee CJ, Park JS (2020). Strategies for optimizing the production of proteins and peptides with multiple disulfide bonds. Antibiotics.

[2] Ahmad M, Hirz M, Pichler H, Schwab H (2014). Protein expression in Pichia pastoris: recent achievements and perspectives for heterologous protein production. Applied microbiology and biotechnology.

[3] Stovicek V, Holkenbrink C, Borodina I (2017). CRISPR/Cas system for yeast genome engineering: advances and applications. FEMS yeast research.

[4] Werten MW, Eggink G, Stuart MAC, de Wolf FA (2019). Production of protein-based polymers in Pichia pastoris. Biotechnology advances.

[5] Berlec A, Štrukelj B (2013). Current state and recent advances in biopharmaceutical production in Escherichia coli, yeasts and mammalian cells. Journal of industrial microbiology and biotechnology.

[6] Looser V, Bruhlmann B, Bumbak F, Stenger C, Costa M, Camattari A, Fotiadis D, Kovar K (2015). Cultivation strategies to enhance productivity of Pichia pastoris: a review. Biotechnology advances.

[7] Steffen KK, Dillin A (2016). A ribosomal perspective on proteostasis and aging. Cell metabolism.

[8] Anisimova AS, Alexandrov AI, Makarova NE, Gladyshev VN, Dmitriev SE (2018). Protein synthesis and quality control in aging. Aging (Albany NY).

[9] Kaeberlein M, Burtner CR, Kennedy BK (2007). Recent developments in yeast aging. PLoS genet.

[10] Dahiya R, Mohammad T, Alajmi MF, Rehman M, Hasan GM, Hussain A, Hassan M (2020). Insights into the Conserved Regulatory Mechanisms of Human and Yeast Aging. Biomolecules.

[11] Kaeberlein M, Kirkland KT, Fields S, Kennedy BK (2005). Genes determining yeast replicative life span in a long-lived genetic background. Mechanisms of ageing and development.

[12] McCormick MA, Delaney JR, Tsuchiya M, Tsuchiyama S, Shemorry A, Sim S, Chou ACZ, Ahmed U, Carr D, Murakami CJ, Schleit J, Sutphin GL, Wasko BM, Bennett CF, Wang AM, Oslen B, Beyer RP, Bammler TK, Prunkard D, Johnson SC, Pennypacker JK, An E, Anies A, Castanza AS, Choi E, Dang E, Enerio S, Fletcher M, Fox L, Goswami S, Higgins SA, Holmberg MA, Hu D, Hui J, Jelic M, Jeong KS, Johnston E, Kerr EO, Kim J, Kim D, Kirkland K, Klum S, Kotireddy S, Liao E, Lim M, Lin MS, Lo WC, Lockshon D, Miller HA, Moller RM, Muller B, Oakes J, Pak DN, Peng ZJ, Pham KM, Pollard TG, Pradeep P, Pruett D, Rai D, Robison B, Rodriguez AA, Ros B, Sage M, Singh MK, Smith ED, Snead K, Solanky A, Spector BL, Steffen KK, Tchao BN, Ting MK, Wende HV, Wang D, Welton KL, Westman EA, Brem RB, Liu XG, Suh Y, Zhou Z, Kaeberlein M, Kennedy BK (2015). A comprehensive analysis of replicative lifespan in 4,698 single-gene deletion strains uncovers conserved mechanisms of aging. Cell metabolism.

[13] Chatterjee K, Nostramo RT, Wan Y, Hopper AK (2018). tRNA dynamics between the nucleus, cytoplasm and mitochondrial surface: location, location, location. Biochimica et biophysica acta (BBA)-gene regulatory mechanisms.

[14] Kenyon C (2001). A conserved regulatory system for aging. Cell.

[15] Osiewacz HD (2002). Genes, mitochondria and aging in filamentous fungi. Ageing research reviews.

[16] Sambrook J, Russell DW, Maniatis T (2001). Molecular Cloning. Cold Spring Habour Laboratory Press: New York.

[17] Rodriguez-Tudela J, Barchiesi F, Bille J, Chryssanthou E, Cuenca-Estrella M, Denning D, Donnelly JP, Dupont B, Fegeler W, Moore C, Richaedson M (2003). Method for the determination of minimum inhibitory concentration (MIC) by broth dilution of fermentative yeasts. Clinical microbiology and infection.

[18] Macauley-Patrick S, Fazenda ML, McNeil B, Harvey LM (2005). Heterologous protein production using the Pichia pastoris expression system. Yeast.

[19] Zhang AL, Luo JX, Zhang TY, Pan YW, Tan YH, Fu CY, Tu FZz (2009). Recent advances on the GAP promoter derived expression system of Pichia pastoris. Molecular biology reports.

[20] Shojaosadati SA, Varedi Kolaei SM, Babaeipour V, Farnoud AM (2008). Recent advances in high cell density cultivation for production of recombinant protein. Iranian journal of biotechnology.

[21] Sagmeister P, Wechselberger P, Herwig C (2012). Information processing: rate-based investigation of cell physiological changes along design space development. PDA journal of pharmaceutical science and technology.

[22] Xiao A, Zhou X, Zhou L, Zhang Y (2006). Improvement of cell viability and hirudin production by ascorbic acid in Pichia pastoris fermentation. Applied microbiology and biotechnology.

[23] Pekarsky A, Veiter L, Rajamanickam V, Herwig C, Grünwald-Gruber C, Altmann F, Spadiut O (2018). Production of a recombinant peroxidase in different glyco-engineered Pichia pastoris strains: a morphological and physiological comparison. Microbial cell factories.

[24] Minor RK, Allard JS, Younts CM, Ward TM, de Cabo R (2010). Dietary interventions to extend life span and health span based on calorie restriction. Journals of gerontology series A: biomedical sciences and medical sciences.

[25] Polymenis M, Kennedy BK (2012). Chronological and replicative lifespan in yeast: do they meet in the middle?. Cell cycle.

[26] Tavernarakis N (2008). Ageing and the regulation of protein synthesis: a balancing act?. Trends in cell biology.

[27] Tavernarakis N (2007). Protein synthesis and aging: eIF4E and the soma vs germline distinction. Cell cycle.

[28] Sampaio-Marques B, Ludovico P (2018). Linking cellular proteostasis to yeast longevity. FEMS yeast research.

[29] Nyström T, Liu B (2014). Protein quality control in time and space–links to cellular aging. FEMS yeast research.

[30] Gonskikh Y, Polacek N (2017). Alterations of the translation apparatus during aging and stress response. Mechanisms of ageing and development.

[31] Saarikangas J, Barral Y (2015). Protein aggregates are associated with replicative aging without compromising protein quality control. Elife.

[32] Markina-Iñarrairaegui A, Etxebeste O, Herrero-García E, Araújo-Bazán L, Fernández-Martínez J, Flores JA, Osmani SA, Espeso EA (2011). Nuclear transporters in a multinucleated organism: functional and localization analyses in Aspergillus nidulans. Molecular biology of the cell.

[33] Azizi A, SharifiRad A, Enayati S, Azizi M, Bayat M, Khalaj V (2020). Absence of AfuXpot, the yeast Los1 homologue, limits Aspergillus fumigatus growth under amino acid deprived condition. World journal of microbiology and biotechnology.

[34] McFarland MR, Keller CD, Childers BM, Adeniyi SA, Corrigall H, Raguin A, Romano MC, Stansfield I (2020). The molecular aetiology of tRNA synthetase depletion: induction of a GCN4 amino acid starvation response despite homeostatic maintenance of charged tRNA levels. Nucleic acids research.

[35] Buchetics M, Dragosits M, Maurer M, Rebnegger C, Porro D, Sauer M, Gasser B, Mattanovich D (2011). Reverse engineering of protein secretion by uncoupling of cell cycle phases from growth. Biotechnology and bioengineering.

[36] Puxbaum V, Gasser B, Mattanovich D (2016). The bud tip is the cellular hot spot of protein secretion in yeasts. Applied microbiology and biotechnology.

[37] Dumont FJ, Su Q (1996). Mechanism of action of the immunosuppressant rapamycin. Life sciences.

[38] Doi A, Fujimoto A, Sato S, Uno T, Kanda Y, Asami K, Tanaka Y, Kita A, Satoh R, Sugiura R (2015). Chemical genomics approach to identify genes associated with sensitivity to rapamycin in the fission yeast Schizosaccharomyces pombe. Genes cells.

[39] McKeehan W, Hardesty B (1969). The mechanism of cycloheximide inhibition of protein synthesis in rabbit reticulocytes. Biochemical and biophysical research communications.

[40] Schneider-Poetsch T, Ju J, Eyler DE, Dang Y, Bhat S, Merrick WC, Green R, Shen B, Liu JO (2010). Inhibition of eukaryotic translation elongation by cycloheximide and lactimidomycin. Nature chemical biology.

